# An example of the utility of genomic analysis for fast and accurate clinical diagnosis of complex rare phenotypes

**DOI:** 10.1186/s13023-017-0582-8

**Published:** 2017-02-07

**Authors:** Polona Le Quesne Stabej, Chela James, Louise Ocaka, Mehmet Tekman, Stephanie Grunewald, Emma Clement, Horia C. Stanescu, Robert Kleta, Deborah Morrogh, Alistair Calder, Hywel J. Williams, Maria Bitner-Glindzicz

**Affiliations:** 1 Genetics and Genomic Medicine, UCL Great Ormond Street Institute of Child Health, 30 Guilford Street, London, WC1N 1EH UK; 2 Division of Medicine, UCL, London, UK; 3 Department of Paediatric Metabolic Medicine, Great Ormond Street Hospital for Children NHS Foundation Trust, London, UK; 4 North East Thames Regional Genetics Service, Great Ormond Street Hospital for Children NHS Foundation Trust, London, UK; 5 North East Thames Regional Genetics Laboratory, London, UK; 6 Radiology Department, Great Ormond Street Hospital for Children NHS Foundation Trust, London, UK

**Keywords:** Next generation sequencing, Autosomal recessive non-syndromic hearing loss, *PDZD7*, Osteogenesis imperfecta, Mosaicism, *COL1A1* C-propeptide cleavage site

## Abstract

**Background:**

We describe molecular diagnosis in a complex consanguineous family: four offspring presented with combinations of three distinctive phenotypes; non-syndromic hearing loss (NSHL), an unusual skeletal phenotype comprising multiple fractures, cranial abnormalities and diaphyseal expansion, and significant developmental delay with microcephaly. We performed Chromosomal Microarray Analysis on the offspring with either the skeletal or developmental delay phenotypes, and linkage analysis and whole exome sequencing (WES) on all four children, parents and maternal aunt.

**Results:**

Chromosomal microarray and FISH analysis identified a *de novo* unbalanced translocation as a cause of the microcephaly and severe developmental delay. WES identified a NSHL-causing splice variant in an autosomal recessive deafness gene *PDZD7* which resided in a linkage region and affected three of the children. In the two children diagnosed with an unusual skeletal phenotype, WES eventually disclosed a heterozygous *COL1A1* variant which affects C-propetide cleavage site of *COL1*. The variant was inherited from an apparently unaffected mosaic father in an autosomal dominant fashion. After the discovery of the *COL1A1* variant, the skeletal phenotype was diagnosed as a high bone mass form of osteogenesis imperfecta.

**Conclusions:**

Next generation sequencing offers an unbiased approach to molecular genetic diagnosis in highly heterogeneous and poorly characterised disorders and enables early diagnosis as well as detection of mosaicism.

**Electronic supplementary material:**

The online version of this article (doi:10.1186/s13023-017-0582-8) contains supplementary material, which is available to authorized users.

## Background

Molecular genetic diagnosis is currently undergoing a considerable transformation with the implementation of next generation sequencing (NGS). The NGS techniques, currently still imperfect, are becoming more accurate as well as affordable, and innovative approaches are being developed to address NGS limitations such as detection of copy number variants (CNV) and structural rearrangements [1]. With NGS generating whole exome (WES) and whole genome sequences (WGS), data management, variant interpretation and genetic counselling is often challenging.

Non-syndromic sensorineural hearing loss (NSHL) is characterised by a high degree of genetic heterogeneity which makes genetic diagnosis exceedingly difficult using traditional Sanger sequencing techniques. At present, 64 genes are implicated in the autosomal recessive form, 35 in autosomal dominant and 4 in the X-linked form [2]. The majority of NSHL causing variants are rare and unique for individual families, with a few notable exceptions.

Bone fragility with fractures in infancy or early childhood has been reported in over 100 genetic disorders from skeletal dysplasias and inborn errors of metabolism to congenital insensitivity to pain [3]. The most common genetic form of bone fragility is osteogenesis imperfecta (OI). If relying solely on clinical evaluation, the diagnosis of OI, which is phenotypically and genetically heterogeneous, might sometimes be missed either due to mild/underdeveloped phenotype at the age of evaluation or atypical presentation [3]. Molecular genetic diagnosis is therefore instrumental for early and accurate clinical diagnosis. *COL1A1* and *COL1A2* genes, which are responsible for more than 90% of all OI cases, are large with over 50 exons, making genetic sequencing for OI diagnosis time consuming and expensive. While biochemical studies, connective tissue and skeletal dysplasia panels are only helpful in some cases, whole exome and genome sequencing enable efficient, unbiased screening of all known genes involved in bone fragility as well as having the potential for novel gene discovery.

Here we report on a consanguineous family segregating sensorineural hearing loss, an unusual skeletal phenotype and microcephaly with significant developmental delay. We describe detailed molecular investigation involving Chromosomal Microarray (CMA), linkage analysis and whole exome sequencing (WES), which led to molecular genetic diagnosis of all three phenotypes.

## Methods

### Patients

The family is a consanguineous family of Pakistani origin; parents are 1st cousins and the mother’s parents were also 1st cousins.

**Fig. 1:**
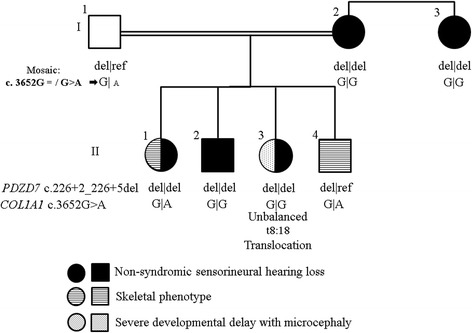
Family pedigree showing the genotypes segregating with the non-syndromic hearing loss, skeletal phenotype and developmental delay with microcephaly

The father (I-1) is healthy, the mother (I-2) and mother’s sister (I-3) have non-syndromic hearing loss (NSHL). The first child (II-1) has NSHL and an unusual skeletal phenotype, the 2nd child (II-2) has NSHL only, the 3rd child (II-3) was born with microcephaly, deep set eyes, global developmental delay in addition to NSHL and the 4th child (II-4) shares the skeletal phenotype of sibling II-1 only (Fig. 1). The 1st, 2nd and 3rd children (II-1, II-2, II-3) failed the newborn hearing screen; the 4th child (II-4) passed the newborn hearing screen.

#### Non-syndromic hearing loss (NSHL) phenotype

The mother (I-2), mother’s sister (I-3), first and second child (II-1 and II-2) have moderate congenital sensorineural hearing loss. The third child (II-3) was born with severe deafness. The fourth child (II-4) passed the newborn screen as well as subsequent visual enforcement audiometry at 1 year. He has normal language development. The father has normal hearing. The mother also has four hearing impaired siblings and four siblings with normal hearing (Fig. 1). Hearing loss is non-progressive and stable. None of these adults have complained of any visual symptoms and one hearing impaired sibling has been tested and found to have a normal electroretinogram (ERG).

#### Skeletal phenotype

**Fig. 2:**
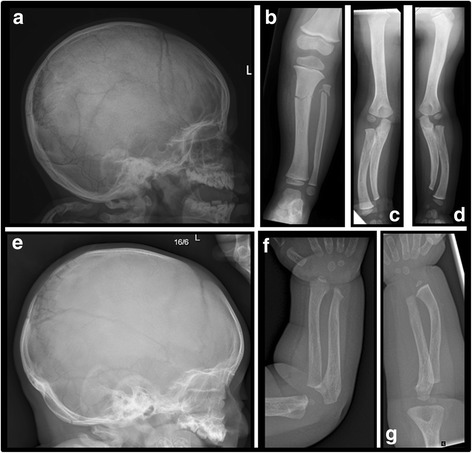
Child II-1. **a** Lateral skull radiograph aged 2 years (a frontal view was not performed at this time). The anterior fontanel is patent. There are multiple Wormian bones around the lambda. **b** Child II-1. Radiograph of left tibia and fibula aged 2 years. There are transverse fractures of the tibia and fibula. The tibia is undermodelled. Bone density appears increased. **c**-**d** Child II-1. Radiographs of the right and left upper limbs aged 2 years. There is pronounced ulnar shortening particularly distally, with varus bowing of the forearm bones. **e**-**g** Child II-4. Lateral skull radiograph demonstrates multiple Wormian bones and radiographs of the forearms aged 11 months ulnar shortening

1st child (II-1): radiographic assessments were made between ages of 23 months and 8 years, including two skeletal surveys. The 1st child sustained fractures of the left 4th and 5th proximal phalanges aged 23 months, and of the left tibial and fibular diaphyses 1 month later when she fell from her cot. There was no evidence of blue sclerae. At the age of 4 years teeth showed dental caries but no dentinogenesis imperfecta. She was also noted at age 6 years to have pars defects of the 5th lumbar vertebra with grade 1 spondylolisthesis. On skeletal survey (Fig. 2a-d), the following radiological features were observed: abnormal Wormian bones with persistent anterior fontanelle at aged 2; shortening of the ulnae with varus bowing of the radii, and radial head dislocation from age 5; diaphyseal expansion (undermodelling) of the tubular bones, particularly the ribs and short tubular bones; abnormal coarse trabecular pattern in short tubular bones; no radiological evidence of osteopenia or vertebral body fractures. Bone density assessment by lumbar spine DEXA at aged 5 was normal (BMAD z-score 0.45), but was significantly increased at ages 6 and 7 (BMAD z z-scores 2.39 and 2.63 respectively).

4th child (II-4): radiographic assessments were made between the ages of 10 days and 19 months, including a skeletal survey (Fig. 2e-g). He sustained fractures of the right humerus perinatally and left femur age 6 months. At age 11 months, multiple Wormian bones and shortening of the ulnae were apparent. Diaphyseal expansion of the ribs and short tubular bones, with abnormal trabeculation, were not apparent at birth, but these features were starting to appear aged 19 months. DEXA z-scores are not available before the age of 5.

The key shared features in this sibling pair are: bony fragility in first 2 years of life; abnormal Wormian bone pattern with late fontanelle closure; mesomelic upper limb shortening with short ulna and bowed radius; undermodelling of tubular bones, particularly ribs and hands, with abnormal trabeculation in the hands; no osteopenia.

Informed consent for the study was obtained from all participants or their parents and genetic studies were approved by the Bloomsbury National Research Ethics Service (Reference number 08/H0713/82). Genomic DNA of the proband and family members was extracted from peripheral blood by standard methods.

#### Developmental delay and microcephaly phenotype

The third child was born at 38 weeks with a birthweight of 2.807 kg following a normal pregnancy and delivery by forceps. She failed the newborn hearing screen and was subsequently fitted with hearing aids. During the first year of life she was diagnosed with global developmental delay and all developmental milestones were delayed. There were initial feeding and swallowing difficulties which resolved after a few months. She sat independently at 1 year. At the age of 16 months her head was on the 0.4th centile, weight was just above the 9th centile and height was just below the 0.4th centile. She learned to stand at 20 months, which was when she was last seen. She was observed to have deep-set eyes, a prominent nasal bridge, microbrachycephaly and large ears. She has a structurally normal heart, hypermetropia and a normal electroretinogram. Biochemical investigations including plasma amino acids, vacuolated lymphocytes, isoelectric focussing of transferrins, very long chain fatty acids, lactate, white cell enzymes, calcium, phosphate and parathyroid hormone levels as well as urea and electrolytes and liver function tests were all normal.

### Microarrays and linkage analysis

The parents and offspring with the skeletal (1st and 4th child) or developmental delay (3rd child) phenotypes were subjected to routine Chromosomal Microarray Analysis (CMA) either by Comparative Genomic Hybridisation (CGH) using Roche Nimblegen 135 K microarray or by cytogenomic SNP microarray using Affymetrix Cytoscan 750 K as per manufacturer’s instructions. Multipoint parametric linkage studies of all seven members of the family (Fig. 1) was performed using the HumanCytoSNP-12v2-1_H Beadarray (Illumina, Inc, San Diego, CA) as per the manufacturer’s instructions as published previously [4]. A recessive transmission model with complete penetrance and a disease allele frequency of 0.0001 was used for linkage analysis for deafness and for skeletal dysplasia initially.

### Whole exome and Sanger sequencing

Whole exome capture was performed with Agilent SureSelect version 4 (Santa Clara, CA), according to manufacturer’s protocol on all seven family members. Enriched libraries were sequenced on the Illumina (San Diego, CA) HiSeq2000. Sequencing reads passing quality filters were aligned to the reference genome build GRGh37/hg19 using Burrows-Wheeler Aligner (BWA) algorithm and for variant calling we applied GATK [5] base quality score recalibration, indel realignment, duplicate removal, and performed SNP and INDEL discovery and genotyping using standard hard filtering parameters or variant quality score [6].

The variant annotation and interpretation analyses were generated through the use of Ingenuity® Variant Analysis™ software version 3.1.20140902 from Ingenuity Systems. For the recessive model, homozygous/compound heterozygous variants shared between affected individuals were retained; for a dominant model, we retained heterozygous variants in common between affected individuals. Intronic and exonic synonymous variants were filtered out; exonic and splice variants (up to seven base pairs into intron or predicted pathogenic on MaxEntScan) with a public databases (ExAC, 1000 Genomes and ESP Exomes) frequency <0.1% (NSHL phenotype) and <0.01% (skeletal phenotype) were retained. Separate filtering steps were applied for NSHL and skeletal phenotypes.

Disease causing variants (*PDZD7* and *COL1A1*) were validated by Sanger sequencing. Primers were developed using Primer3 software (see Additional file 1 for primer sequences).

### RNA isolation and PDZD7 RT-PCR

Blood samples from parents and all four children were collected into Paxgene™ RNA tubes and RNA from whole blood was extracted according to manufacturer’s protocol using the PAXgene Blood RNA System (PreAnalytiX, Hombrechtikon, CH). Synthesis of cDNA was carried out using Omniscript™ RT (Qiagen, Santa Clarita, CA) system according to supplier’s protocol. PCR primer sequences used to amplify *PDZD7* exons 1–15 are listed in Additional file 2.

## Results

### Chromosomal Microarray Analysis (CMA)

Copy number variants (CNV) identified by CMA were analysed with respect to gene content, presence on public databases (DECIPHER, DGV) and whether they segregated with one of the phenotypes. CMA identified monosomy 8p23 with a loss of material of approximately 10,6 Mb between 8:176,569 and 10,781,689 and partial trisomy 18 with a gain of approximately 7.9 Mb between co-ordinates 18:141,489-8,022,950, suggesting a *de novo* unbalanced translocation between chromosomes 8 and 18 as the cause of severe developmental delay with microcephaly in the 3rd child (II-3). More than 100 genes are either deleted or duplicated by the unbalanced translocation and are likely to contribute to the phenotype of the patient. These include three DDG2P genes on chromosome 18 (*TGIF1*, *LAMA1*, *SMCHD1*) and three on chromosome 8 (*CLN8*, *MCPH1*, *FBOX25*) [7, 8]. Deletion of this region of chromosome 8 has been reported in association with developmental delay and microcephaly. Duplication of a similar region of chromosome 18 has been described associated with developmental delay and behavioural problems. The translocation was not observed in any other family member and was thought to be responsible for the significant developmental delay and microcephaly phenotypes, but not the NSHL. No further CNVs were identified that could explain any of the clinical phenotypes.

### Linkage analysis

Multipoint parametric linkage analysis showed six regions with significant linkage (logarithm of odds scores, LOD > 3.0) to the NSHL phenotype using a recessive model; i.e. on chromosomes 3, 4, 7, 10 (two regions) and 13 (Additional files 3 and 4).

Linkage analysis though failed to reveal any regions significantly linked to the skeletal phenotype (Additional file 5) using a recessive model. Despite a lack of significant LOD scores in the skeletal phenotype analysis, we looked for homozygous and compound heterozygous variants in the linkage intervals with LOD scores greater than 2 (i.e. between 2.0172 and 2.0556 on chromosomes 5, 9, 14 and 16) shared between the two sibs affected with skeletal phenotype (Additional file 5). There were no rare variants (less frequent than 1% in public databases) in the exonic or splice (up to +/−7 into an intron) regions shared between the two sibs and not shared by skeletal phenotype unaffected sibs. We also manually inspected the BAM files for potential larger deletions in these regions and failed to find any.

### Whole Exome Sequence (WES) analysis

Whole exome sequence with mean 70× coverage was generated for the parents, all four children and the mother’s sister. Separate analyses were performed for the NSHL and skeletal phenotypes.

For the NSHL phenotype, only the *PDZD7* NM_001195263.1:c.226 + 2_226 + 5del (hg19.chr10:g.102789746_102789749del) variant passed the genetic filter (Fig. 1). It also resided in the linkage region on chromosome 10 with a significant LOD score of 3.6 (Additional files 3 and 4). The *PDZD7* c.226 + 2_226 + 5del variant is a homozygous deletion of four base pairs at the exon 2 - intron 2 junction. The deletion affects the invariant splice site and is therefore predicted to affect splicing. The *PDZD7* c.226 + 2_226 + 5del was not found in any public databases (ExAC, ESP Exomes, 1000 Genomes and gnomAD) [9].

**Fig. 3:**
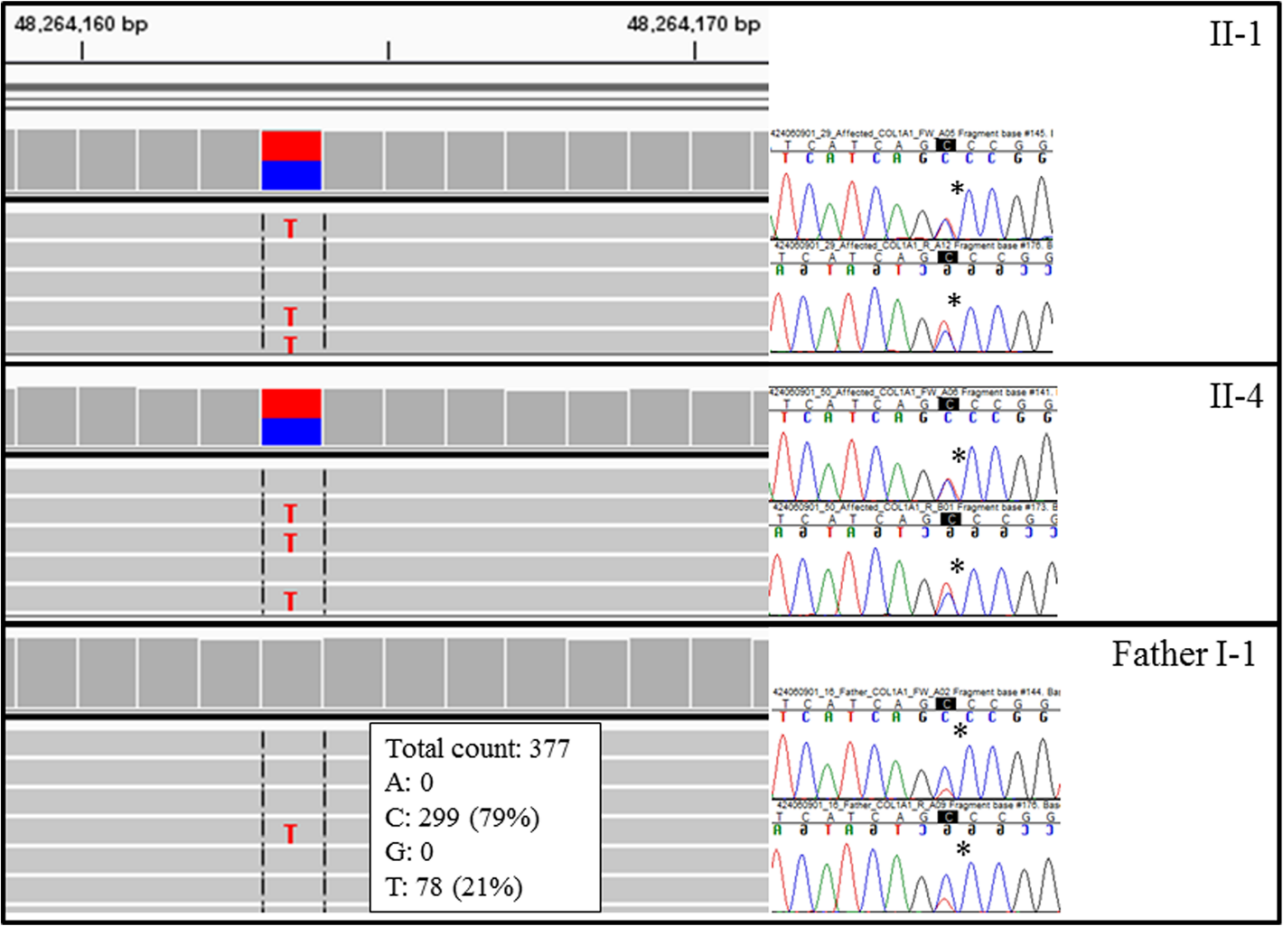
BAM and Sanger sequence traces showing *COL1A1* c.3652G > A variant: heterozygous in the two affected children (II-1, II-4) with skeletal phenotype and mosaic in the father (I-1)

WES analysis to find the variant causing the skeletal phenotype proved challenging; in line with the absence of linkage regions, we found no potential recessive disease causing variants shared between the two affected sibs with the skeletal phenotype. This led us to consider an autosomal dominant scenario, with the disease-causing variant inherited from a parent who was either a gonosomal or gonadal mosaic. There were no *de novo* variants shared between the two affected sibs which were absent in parents or other unaffected sibs; pure gonadal mosaicism was therefore excluded. In order to investigate gonosomal mosaicism, we looked for variants shared between affected sibs, but not unaffected sibs, and possibly present in either parent. There were nine rare (<0.01% in public databases) good quality heterozygous exonic/splice variants shared between the two sibs affected with the skeletal phenotype and not called in unaffected sibs, but present in either one of the parents. We assessed the proportions of reads carrying the alternate allele (allele fraction – AF) in all nine variants in the parents. While the average allele fraction in all heterozygous variants was 47%, the *COL1A1* NM_000088.3:c.3652G > A (chr17.hg19:48264163C > T) heterozygous variant had an AF of only 21% in the father; i.e. of the 377 aligned paternal reads, 78 carried the alternate allele “T” while 299 carried the reference allele “C”. By contrast, children affected with the skeletal phenotype II-1 and II-4 carried the alternate variant in 49% and 52% of reads, respectively (Figs. 1 and 3). The paternal mosaicism as well as the presence/absence of the *COL1A1* variant in the other family members has been confirmed by Sanger sequencing. *COL1A1* c.3652G > A (p.Ala1218Thr) is not present in any public database (ExAC, ESP Exomes, 1000 Genomes or gnomAD [9]), is conserved in 100 vertebrates and is predicted to be deleterious and disease causing according to SIFT and MutationTaster, respectively.

### PDZD7 cDNA

The 4-nucleotide deletion in *PDZD7* (c.226 + 2_226 + 5delTAGG) is predicted to affect splicing as it abolishes the invariant GT splice site at the exon 2 - intron 2 junction. On the cDNA level, we confirmed the presence of a *PDZD7* product spanning exons 1–2 in the family members affected with NSHL as well as unaffected members, demonstrating that this part of the transcript was expressed at detectable levels in lymphocyte RNA. However, we were not able to amplify *PDZD7* exons 3–17 in individuals affected with NSHL. In the NSHL unaffected heterozygous father, and in normal controls we were able to obtain a product spanning exons 2–6, 5–8 and 13–15, but Sanger sequencing of these products revealed that in the father, only the allele not carrying the c.226 + 2_226 + 5deletion was amplified (ie. only wild-type was expressed). The distinction between the two alleles was possible due to the presence of SNPs rs148746572 residing in *PDZD7* exon 6 and rs547610251 in exon 15; in the whole exome genomic sequence the father is heterozygous for both SNPs, but in the cDNA sequence he is hemizygous (Additional files 6 and 7).

## Discussion

Here we demonstrate the efficiency of WES in solving the underlying molecular diagnoses in a family with complex phenotypes. While conventional Chromosomal Microarray was quick to indicate an unbalanced translocation, with monosomy of 8p23 and partial trisomy of chromosome 18 as the cause of severe developmental delay and microcephaly in the 3rd child, the causes of the NSHL and uncharacterised skeletal phenotypes in the remaining siblings were more challenging. A first-generation inherited hearing loss panel with 55 deafness genes failed to establish genetic diagnosis of NSHL since *PDZD7* had not been associated with NSHL and due to the unusual presentation the skeletal phenotype eluded a definite clinical diagnosis. By the time the 4th child was born, all available family members underwent linkage analysis and whole exome sequencing. As expected (based on strong history of NSHL on the mother’s side, consanguinity as well as multiple affected family members), the genetic cause of NSHL was a variant inherited in an autosomal recessive manner - a homozygous *PDZD7*c.226 + 2_226 + 5del at the exon 2 – intron 2 junction which involves the invariant GT splice site. *PDZD7* gene was initially described as a modifier of the retinal phenotype in Usher syndrome; more recently however, it has been shown to cause autosomal recessive NSHL [10–12].

The *COL1A1*c.3652A > G (p.Ala1218Thr) variant, which was inherited in an autosomal dominant manner from a mosaic father, has previously been described in a mother and son with ‘gnathodiaphyseal dysplasia’ and in two siblings diagnosed as ‘osteogenesis imperfecta with increased bone mineral density’ (BMD) [13, 14]. The two sibs described in our report had no jaw lesions or late tooth eruption as described in the gnathodiaphyseal dysplasia case. The common denominators with the gnathodiaphyseal case were multiple fractures, elevated BMD and late fontanelle closure. Multiple fractures and elevated BMD were also the characteristics of OI with increased BMD described by Cundy et al. But unlike the pediatric patients in our study, the affected sibs described there also had a significant conductive hearing loss and osteosclerosis; this might be due to age-related penetrance [14].

Type I collagen is a triple-helical molecule composed of two alpha-1 chains and one alpha-2 chain, encoded by *COL1A1* and *COL1A2*. After the assembly of the chains into a triple helix, the N- and C-propeptides are cleaved by proteinases. Substitutions in the COL1A1 and COL1A2 cleavage sites block the removal of the C-propeptide tails from type I procollagen and result in incorporation of a molecule with retained C-propeptide. The variant reported here is one of the four conserved C-propeptide cleavage site residues (together with Asp1219 in COL1A1, Ala1119 and Asp1120 in COL1A2) that affects the alanine at position 1218 and is expected to abolish the C-propeptide cleavage site. Interestingly, patients with COL1 cleavage site variants had different clinical presentations, but increased bone mineralisation with multiple fractures was described in most cases (see Table 2 in McInerney-Leo et al. 2015) [13–15]. After the discovery of *COL1A1* c.3652A > G mosaicism in the father, the asymptomatic father was examined radiographically (age 28 years). The hands showed subtle diaphyseal expansion of the metacarpals only but no signs of old fractures. Skull sutures were indistinct, but there was no clear abnormal Wormian bone pattern. An orthopantomogram did not show sclerotic jaw lesions. The ulnae showed minor negative minus variance, within normal limits. Lumbar spine DEXA revealed significantly elevated bone mass with age-matched z-score of 3.6 indicating a sub-clinical phenotype.

## Conclusions

In conclusion, due to clinical and genetic heterogeneity of skeletal and hearing loss disorders as well as a clinical picture in pediatric patients which is not always fully manifest at presentation or which is atypical, early diagnosis is difficult. Whole exome sequence analysis was instrumental in establishing the molecular genetic diagnosis in this family and is sensitive enough to detect mosaicism, if suspected. Although NGS platforms are still evolving and imperfect, they are invaluable at this present time as they offer simultaneous unbiased evaluation of all exonic/genomic variants and are rapidly making their way into clinical genetic diagnostics.

## Additional files

Additional file 1: Genomic primers used for Sanger sequencing of *PDZD7* and *COL1A1* variants. (DOCX 13 kb)
Additional file 2:
*PDZD7* cDNA primers used for Sanger sequencing. (DOCX 14 kb)
Additional file 3: Non-syndromic hearing loss phenotype - linkage plot showing logarithm of odds (LOD) scores across the whole genome. (PDF 15 kb)
Additional file 4: Non-syndromic hearing loss phenotype - linkage plot showing logarithm of odds (LOD) scores on chromosome 10 where *PDZD7* resides. (PDF 12 kb)
Additional file 5: Skeletal phenotype - linkage plot with logarithm of odds (LOD) scores across the whole genome – no regions were found to be significantly linked to the skeletal phenotype. (PDF 16 kb)
Additional file 6:
*PDZD7* haplotypes for the mother (NSHL affected) and father (unaffected) on genomic DNA and mRNA/cDNA. Genomic DNA sequence is based on whole exome sequence (BAM files); cDNA sequences are Sanger sequences of mRNA/cDNA isolated from blood (for primer sequences see Additional file 2). “-│-” indicates there was no amplification in the mother. (PPTX 40 kb)
Additional file 7: Parents’ genomic DNA sequence (BAM) and father’s cDNA Sanger sequence depicting rs148746572 in exon 6 (**a**) and rs547610251 in exon 15 (**b**) with corresponding genomic DNA sequence as seen on BAM file. (PDF 269 kb)
