# Supplementary material for: An example of the utility of genomic analysis for fast and accurate clinical diagnosis of complex rare phenotypes
Source: Orphanet J Rare Dis. 2017 Feb 7;12:24. doi: 10.1186/s13023-017-0582-8 (PMC5297239; doi:10.1186/s13023-017-0582-8)
Supplement: Additional file 1: — Genomic primers used for Sanger sequencing of PDZD7 and COL1A1 variants. (DOCX 13 kb) [file 13023_2017_582_MOESM1_ESM.docx]

| Primer Name | Primer Sequence | Product size (bp) |
| --- | --- | --- |
| PDZD7_ex2+2 DEL_FW | ATGGCCTTCTTTCCCTCCTC | 632 |
| PDZD7_ex2+2 DEL_R | CCTGGAGCTCACACTTCTGA |  |
| COL1A1_A1218T_FW | CCCTCACCACTCTTCCAGTC | 316 |
| COL1A1_A1218T_R | CCCCACTCTCTTCCCTCTCT |  |
