# Supplementary material for: An example of the utility of genomic analysis for fast and accurate clinical diagnosis of complex rare phenotypes
Source: Orphanet J Rare Dis. 2017 Feb 7;12:24. doi: 10.1186/s13023-017-0582-8 (PMC5297239; doi:10.1186/s13023-017-0582-8)
Supplement: Additional file 2: — PDZD7 cDNA primers used for Sanger sequencing. (DOCX 14 kb) [file 13023_2017_582_MOESM2_ESM.docx]

| Primer Name | Primer Sequence | Product size (bp) |
| --- | --- | --- |
| Exon 1_FW | CTGCGGGTGGTTTGACAAG | 359 |
| Exon 2_R | AGCCGTTGTTGCTTCCTTAG |  |
| Exon 2_FW | GCGTCATCCTCATCAACTCC | 1619 |
| Exon 9_R | TATGTCCAGGCGAGGGTAAG |  |
| Exon 2_FW* | GCGTCATCCTCATCAACTCC | 797 |
| Exon 8_R* | GCACGAAGAGACGCTGGAG |  |
| Exon 13_FW | GGAGCTGGAGGCTTTTGAG | 484 |
| Exon 15_R | ACCTGGAGACTTGCCTTGAC |  |
| Exon 14_FW$ | CCAAGCGTCACCTTATCACC | 308 |
| Exon 15_R1$ | AGGGTGCTCTCGGCTCAG |  |

* The PCR product spanning exons 2 to 8 was Sanger sequenced after nested PCR using Exon 2_FW and Exon 8_R primers was performed on the Exon 2_FW and Exon 9_R PCR product.

$ The PCR product spanning exons 13 to 15 was Sanger sequenced after nested PCR using Exon 14_FW and Exon 15_R1 primers was performed on the Exon 13_FW and Exon 15_R PCR product.
