# Supplementary figures and images for: An example of the utility of genomic analysis for fast and accurate clinical diagnosis of complex rare phenotypes
Source: Orphanet J Rare Dis. 2017 Feb 7;12:24. doi: 10.1186/s13023-017-0582-8 (PMC5297239; doi:10.1186/s13023-017-0582-8)

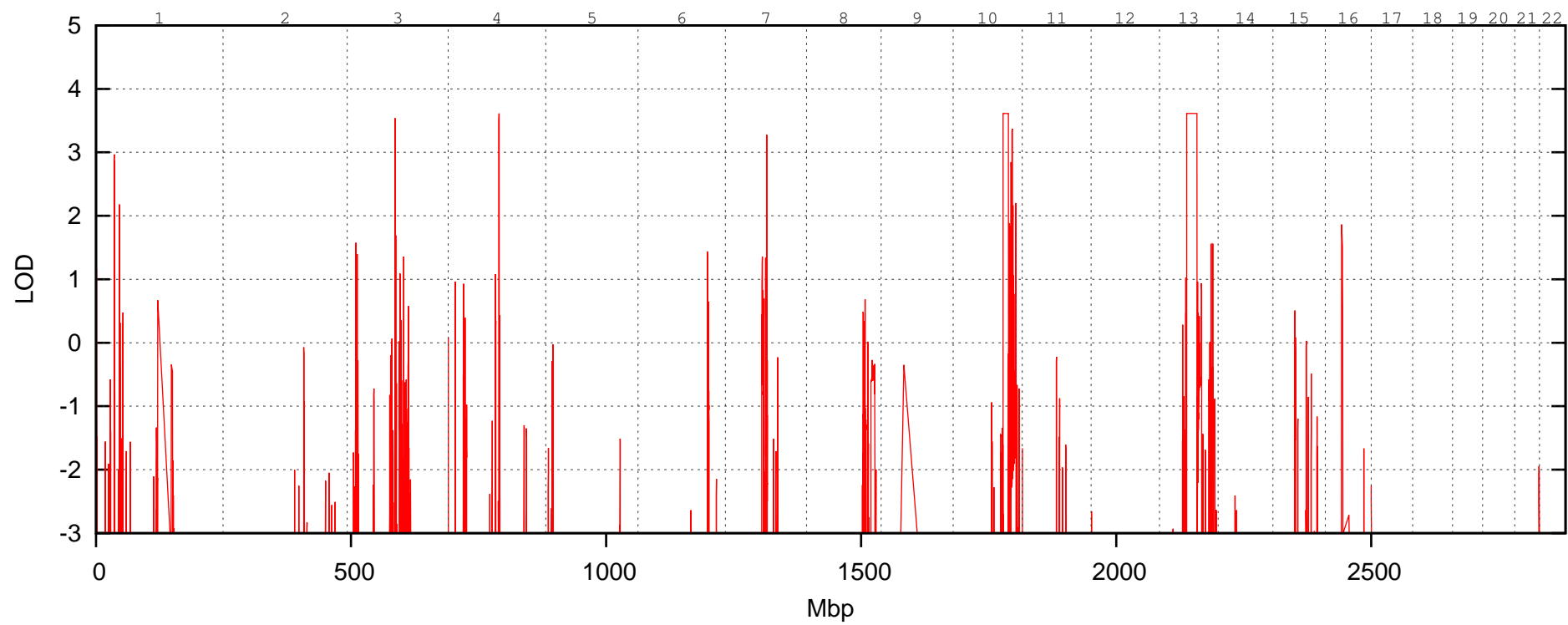

Supplement: Additional file 3: — Non-syndromic hearing loss phenotype - linkage plot showing logarithm of odds (LOD) scores across the whole genome. (PDF 15 kb) [file 13023_2017_582_MOESM3_ESM.pdf]

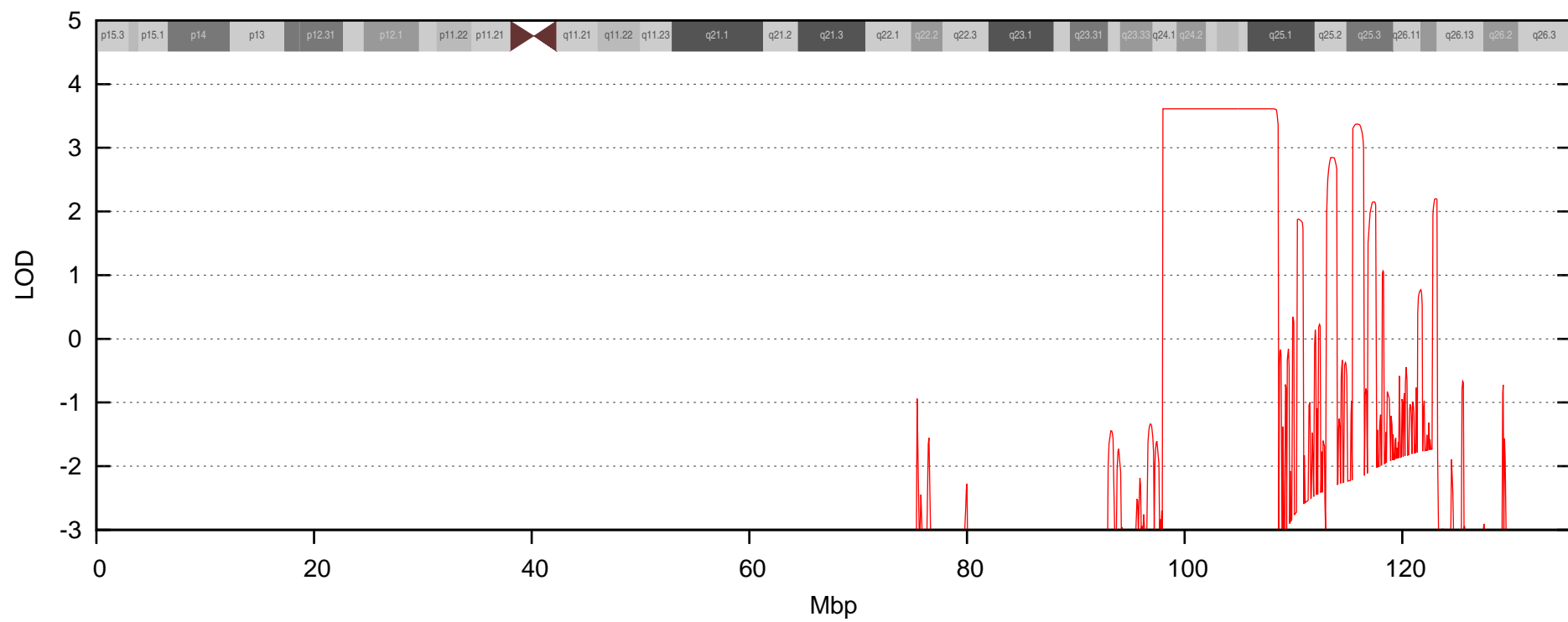

Supplement: Additional file 4: — Non-syndromic hearing loss phenotype - linkage plot showing logarithm of odds (LOD) scores on chromosome 10 where PDZD7 resides. (PDF 12 kb) [file 13023_2017_582_MOESM4_ESM.pdf]

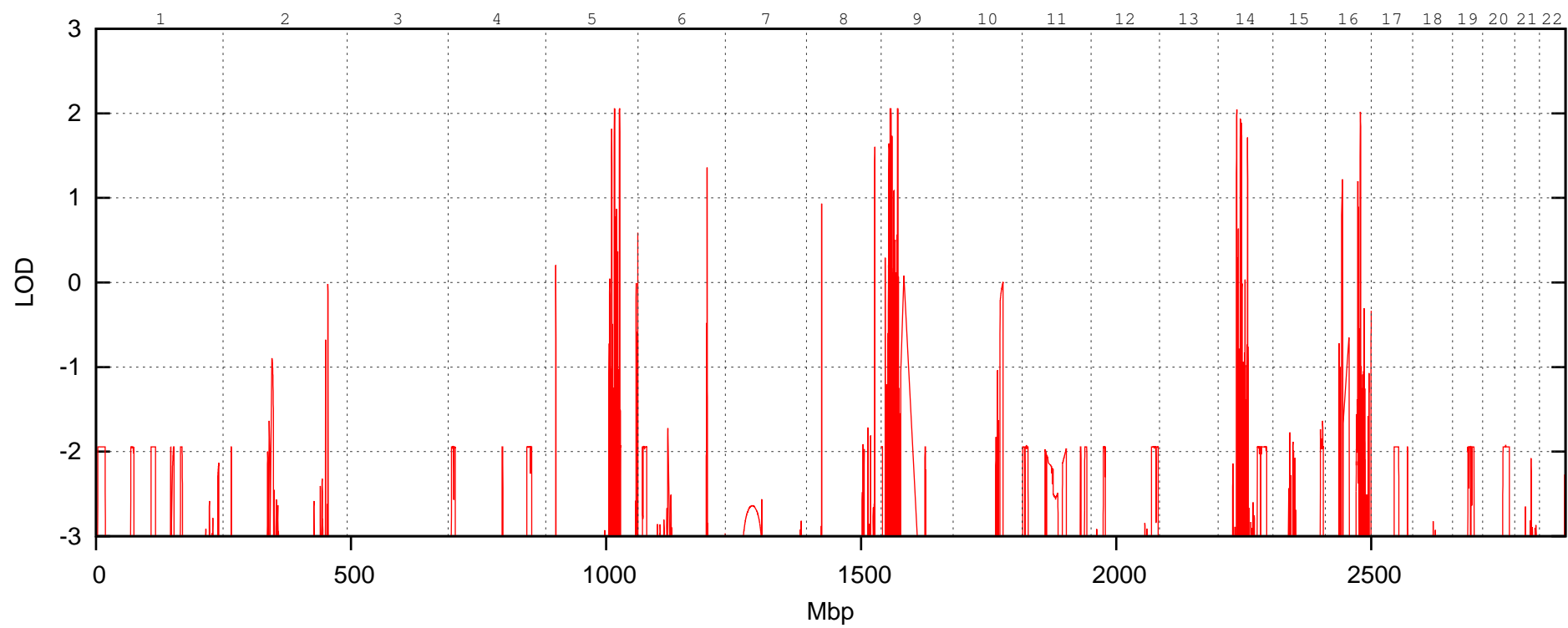

Supplement: Additional file 5: — Skeletal phenotype - linkage plot with logarithm of odds (LOD) scores across the whole genome – no regions were found to be significantly linked to the skeletal phenotype. (PDF 16 kb) [file 13023_2017_582_MOESM5_ESM.pdf]

## Slide 1
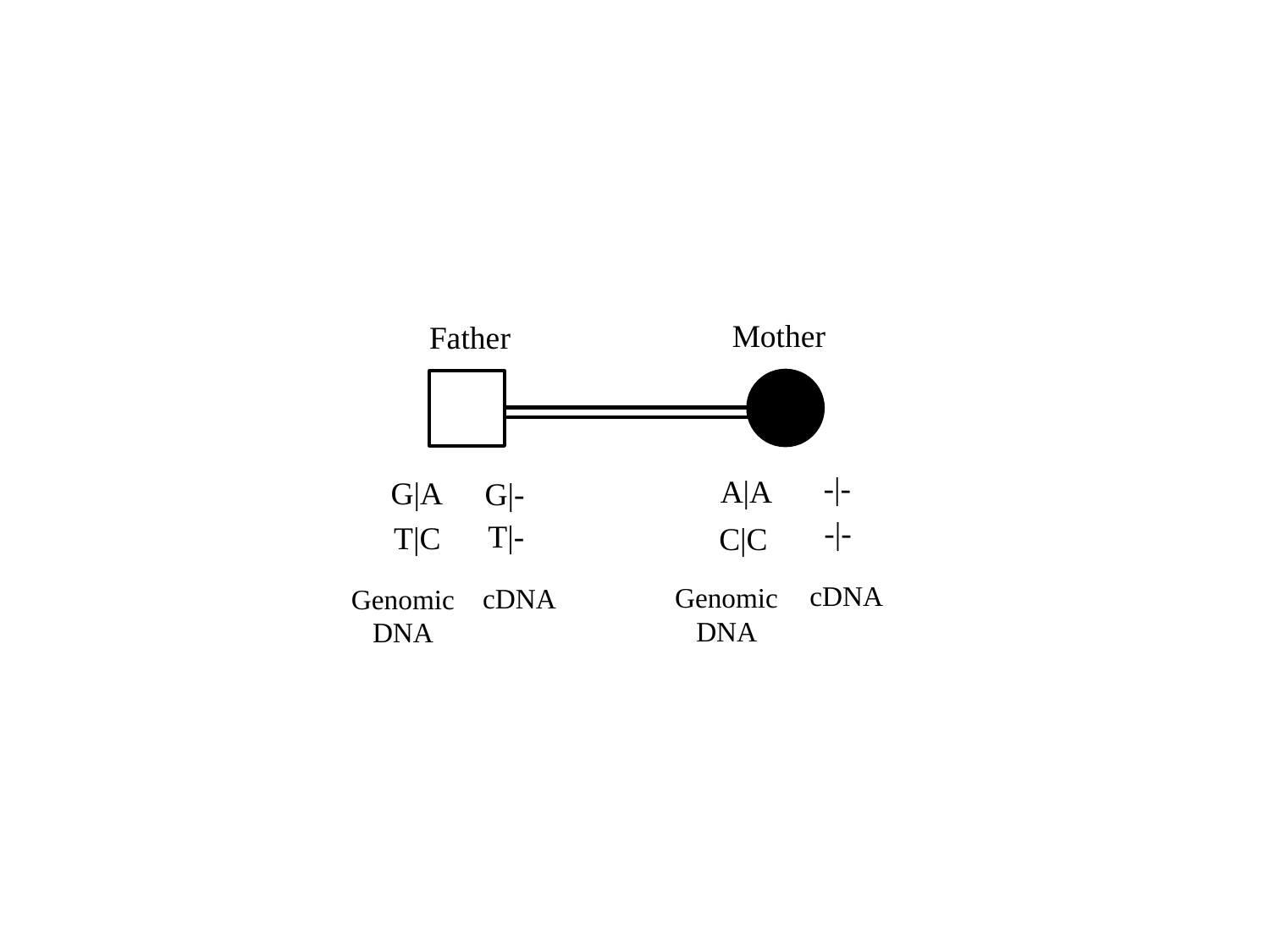

Mother
Father
-|-
A|A
G|A
G|-
-|-
T|-
T|C
C|C
cDNA
Genomic DNA
cDNA
Genomic DNA

Supplement: Additional file 6: — PDZD7 haplotypes for the mother (NSHL affected) and father (unaffected) on genomic DNA and mRNA/cDNA. Genomic DNA sequence is based on whole exome sequence (BAM files); cDNA sequences are Sanger sequences of mRNA/cDNA isolated from blood (for primer sequences see Additional file 2). “-│-” indicates there was no amplification in the mother. (PPTX 40 kb) [file 13023_2017_582_MOESM6_ESM.pptx]
