# Supplementary material for: An example of the utility of genomic analysis for fast and accurate clinical diagnosis of complex rare phenotypes
Source: Orphanet J Rare Dis. 2017 Feb 7;12:24. doi: 10.1186/s13023-017-0582-8 (PMC5297239; doi:10.1186/s13023-017-0582-8)
Supplement: Additional file 7: — Parents’ genomic DNA sequence (BAM) and father’s cDNA Sanger sequence depicting rs148746572 in exon 6 (a) and rs547610251 in exon 15 (b) with corresponding genomic DNA sequence as seen on BAM file. (PDF 269 kb) [file 13023_2017_582_MOESM7_ESM.pdf]

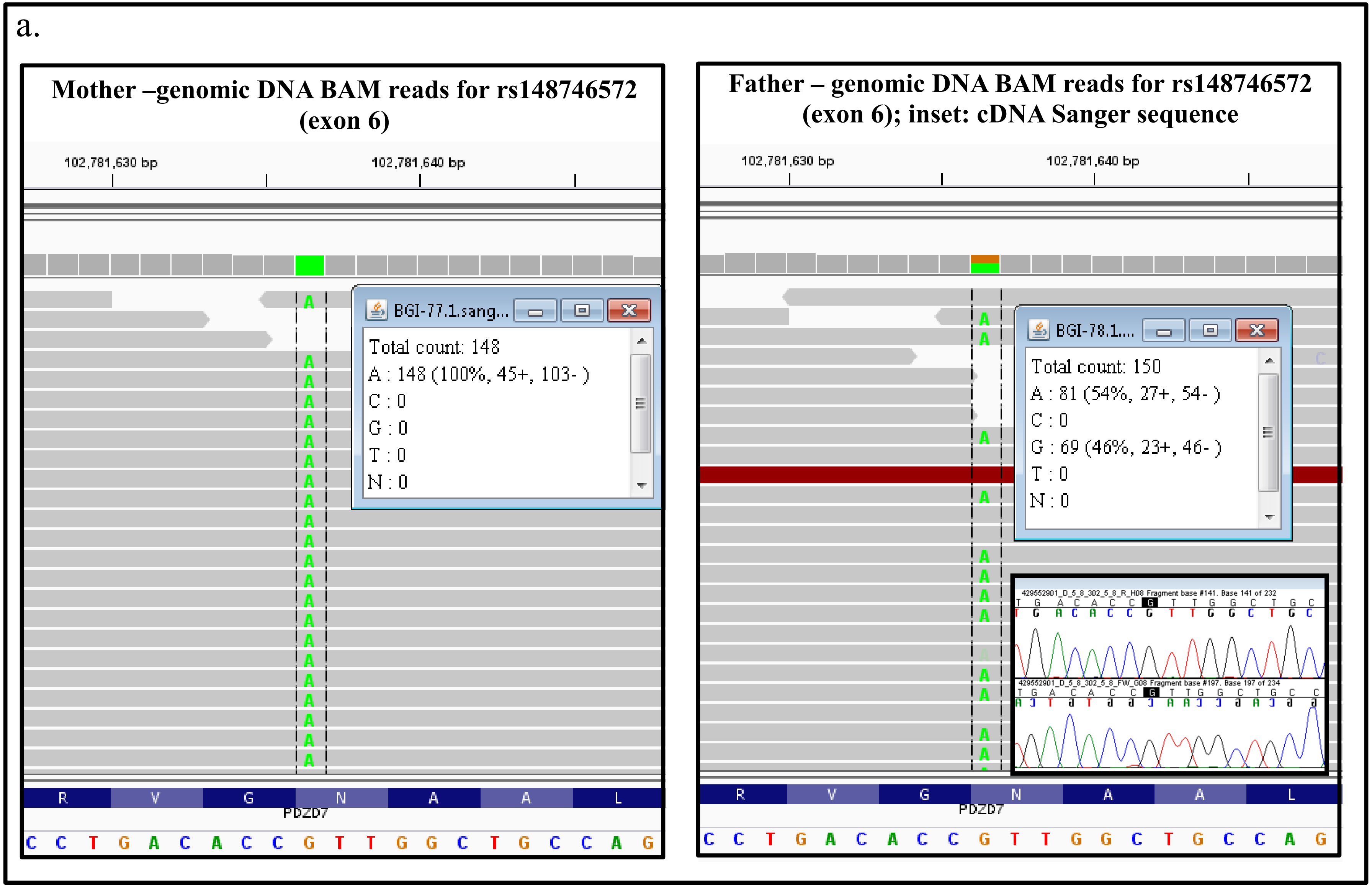
a.
Mother –genomic DNA BAM reads for rs148746572 (exon 6)
102,781,630 bp
102,781,640 bp
BGI-77.1.sang...
Total count: 148
A : 148 (100%, 45+, 103-)
C : 0
G : 0
T : 0
N : 0
R V G N A A L
PDZD7
C C T G A C A C C G T T G G C T G C C A G
Father – genomic DNA BAM reads for rs148746572 (exon 6); inset: cDNA Sanger sequence
102,781,630 bp
102,781,640 bp
BGI-78.1....
Total count: 150
A : 81 (54%, 27+, 54-)
C : 0
G : 69 (46%, 23+, 46-)
T : 0
N : 0
R V G N A A L
PDZD7
C C T G A C A C C G T T G G C T G C C A G

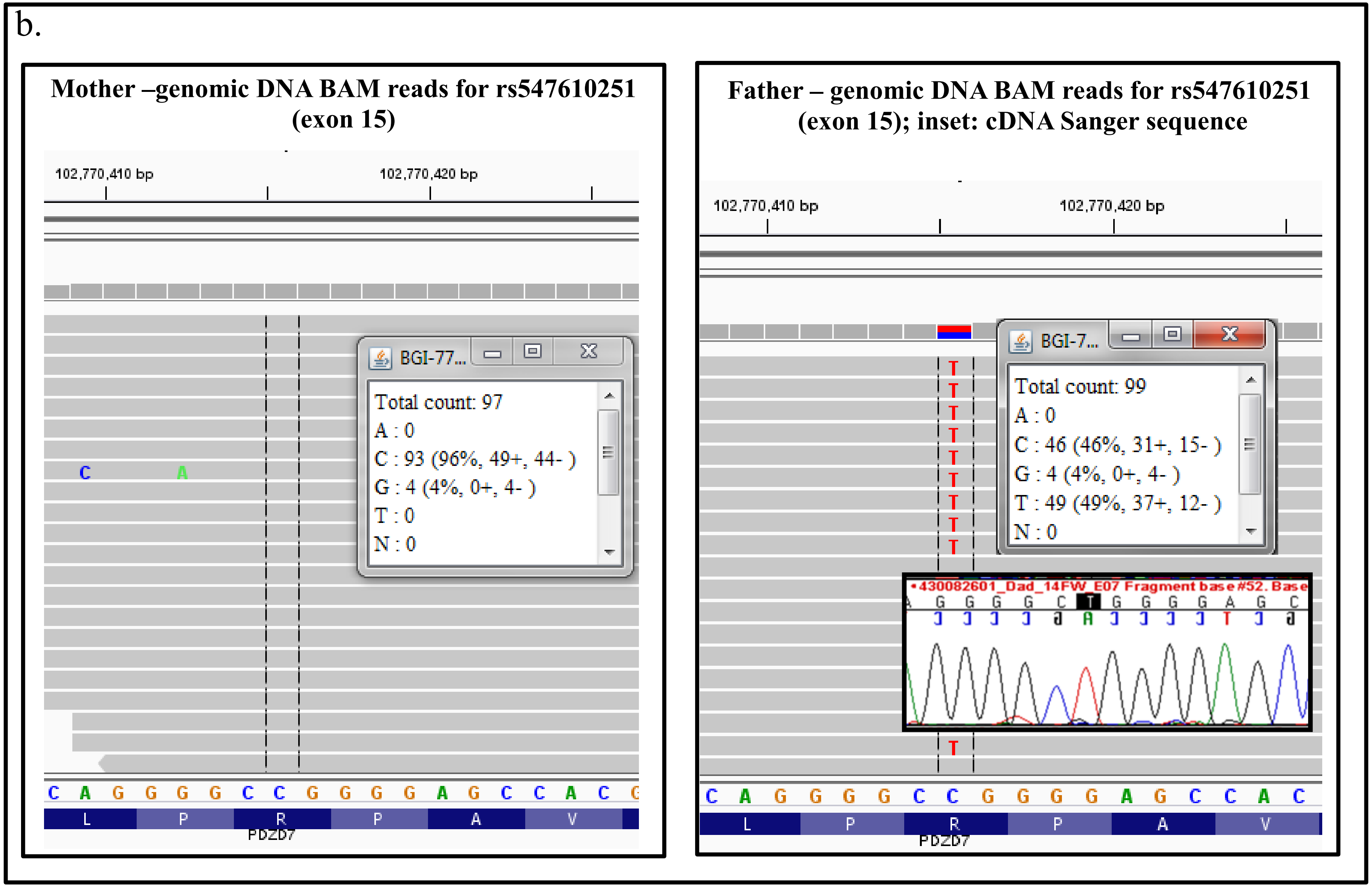
b.
Mother –genomic DNA BAM reads for rs547610251 (exon 15)
102,770,410 bp
102,770,420 bp
BGI-77...
Total count: 97
A : 0
C : 93 (96%, 49+, 44-)
G : 4 (4%, 0+, 4-)
T : 0
N : 0
C A G G G G C C G G G G A G C C A C
L P R P A V
PDZD7
Father – genomic DNA BAM reads for rs547610251 (exon 15); inset: cDNA Sanger sequence
102,770,410 bp
102,770,420 bp
BGI-7...
Total count: 99
A : 0
C : 46 (46%, 31+, 15-)
G : 4 (4%, 0+, 4-)
T : 49 (49%, 37+, 12-)
N : 0
430082601_Dad_14FW_E07 Fragment base #52. Base
C A G G G G C C G G G G A G C C A C
L P R P A V
PDZD7
